# Antimicrobial Properties of Magnesium Open Opportunities to Develop Healthier Food

**DOI:** 10.3390/nu11102363

**Published:** 2019-10-03

**Authors:** Keren Demishtein, Ram Reifen, Moshe Shemesh

**Affiliations:** 1 Department of Food Sciences, Institute for Postharvest Technology and Food Sciences, Agricultural Research Organization, Volcani Center, Rishon LeZion 7528809, Israel; kerend@volcani.agri.gov.il; 2 The Robert H. Smith Faculty of Agriculture, Food and Environment, Institute of Biochemistry, Food Science and Nutrition, The Hebrew University of Jerusalem, Rehovot 7610001, Israel; ram.reifen@mail.huji.ac.il

**Keywords:** healthy food, biofilm, magnesium ions, microbial development, dairy food

## Abstract

Magnesium is a vital mineral that takes part in hundreds of enzymatic reactions in the human body. In the past several years, new information emerged in regard to the antibacterial effect of magnesium. Here we elaborate on the recent knowledge of its antibacterial effect with emphasis on its ability to impair bacterial adherence and formation complex community of bacterial cells called biofilm. We further talk about its ability to impair biofilm formation in milk that provides opportunity for developing safer and qualitative dairy products. Finally, we describe the pronounced advantages of enrichment of food with magnesium ions, which result in healthier and more efficient food products.

## 1. Introduction

Magnesium represents an essential element for life and is ubiquitously found in all organisms. This important cation plays crucial roles as an enzymatic co-factor, as well as it is involved in cellular signaling, and in stabilizing cellular components [1,2]. It is not surprising that magnesium salts are typically associated with positive effects on microbial cells. However, it appears that at elevated doses, for instance at milimolar concentrations, magnesium ions become harmful for prokaryotic cell and therefore may negatively affect important cellular processes [3,4,5,6,7]. Although, some progress has been made in investigating the effect of magnesium ions in different microorganisms, it is still not clear how these vital ions affect the cellular processes in microbial cell. Moreover, the mode of antimicrobial action of magnesium ions remains largely unknown. In the past several years, more information emerged concerning the effect of magnesium on bacterial cells. Consequently, in this mini-review, we summarize recent advances in understanding the antimicrobial properties of magnesium ions with an emphasis on their effect on biofilm formation, which became the biggest microbiological problem in clinical as well as industrial settings. We further discuss the antimicrobial potential of magnesium ions in developing novel approaches towards improving food safety and quality. Finally, we describe new perspectives in developing healthier food for human consumption by its enrichment based on magnesium ions.

## 2. The Antimicrobial Properties of Magnesium

Historically, back in 1915, Professor Pierre Delbet was looking for a solution to cleanse wounds that would replace the traditional antiseptics that damage tissues. After testing several solutions, he found MgCl_2_ solution to be most effective as it had two main advantages—it was not harmful for the tissue and it highly increased leucocyte activity and phagocytosis. Later, he found this solution to be an efficient therapy for various diseases, including diseases related to microorganisms [8]. In the past several years, new interest on this cation arose due to its antimicrobial properties. In several studies, antibiotic activity in the presence of Mg^2+^ ions was found to be more efficient [9,10]. It has been hypothesized that the divalent ions affect the membranes of bacterial cells. One study suggested that the curvature of the bacterial membrane is affected, and eventually the bacteria become more vulnerable, and the antibiotics are more efficient [11]. A different study showed that these cations permeabilize the membranes and cause them to be leakier [5]. Other studies tested the potential antimicrobial effect of coating different surfaces with magnesium or magnesium compounds. These surfaces were found as effective in prevention of bacteria adherence as well as biofilm formation. Some of these compounds were suggested to disrupt the membrane potential, again strengthening the idea that magnesium permeabilizes membranes and eventually cause the bacteria to be more sensitive [6,12,13,14]. Moreover, metal oxide nanoparticles of MgO were tested as antibacterial agents as well [3,6]. Indeed, these particles were found to be effective against yeast and planktonic bacteria as well as against biofilms [3]. In addition, these nanoparticles were found to be of low cytotoxicity and relatively safe. Since biofilm formation is considered as a major problem in the food industry as well as in the biomedical field, a lot of effort is put into dealing with this phenomenon [15,16]. Therefore, the effect of magnesium ions was also tested recently as a potential solution for the biofilm problem.

### 2.1. The Effect of Magnesium on Bacterial Survival and Biofilm Formation

Biofilms are highly structured multicellular communities [17,18,19]. Biofilm formation is a multistage process in which bacterial cells adhere to a surface and/or to each other through production of an extracellular matrix that is typically composed of exopolymeric substances (EPS) such as polysaccharides, proteins, and nucleic acids, which surround and may protect the enclosed bacteria [19,20,21]. They form highly structured multicellular communities that are capable of coordinated and collective behavior [17,18,22]. Bacterial cells in biofilms are characterized by increased resistance to unfavorable environmental conditions, antimicrobial agents, and cleaning chemicals [19,23,24]. It appears that the major source of the contamination of food products is often associated with biofilms on the surfaces of food processing equipment [15,25,26]. Therefore, biofilm formation is considered as a major problem in the food industry [15,26,27].

Several approaches were suggested to deal with biofilm formation in the food industry [15,26]. Environmental factors such as electrolyte concentrations and medium composition were shown to have important impact on biofilm formation [28]. Divalent cations can influence biofilm formation directly through their effect on electro-static interactions and indirectly via physiology-dependent attachment processes by acting as important cellular cations and enzyme cofactors [28,29,30,31]. Due to its potentially important role, the effect of Mg^2+^ ions on biofilm formation has been tested. These ions are crucial for the physiology of bacterial cells, although their excess can be harmful for them. Bacterial cells maintain the tolerable concentrations of Mg^2+^ ions by influx and efflux strategies based on their availability. Bacteria overcome limitations in those ions or respond to excess levels, and this helps to maintain the metal homeostasis within the cell. It appears that Mg^2+^ ions are vital for membrane stabilization and function as a cofactor for diverse enzymatic reactions. Bacteria achieve Mg^2+^ homeostasis by regulating the Mg^2+^ transporters and sensors that coordinate the influx and efflux of Mg^2+^ from the bacterial cell. The Gram-negative bacterium *Salmonella enterica serovar Typhimurium* is one of the best-understood models for explaining the Mg^2+^ homeostasis [32,33]. In *Staphylococcus aureus*, Mg^2+^ was shown to increase the rigidity of cell wall by binding to teichoic acids (TA). TA, bind the positively charged Mg^2+^ ions to mitigate the electrostatic repulsive interactions between the negatively charged neighboring phosphates. In addition, the Mg^2+^ ions start a signaling cascade, which results in expression of biofilm related genes [34]. Furthermore, studies have shown that Mg^2+^ ions have varying effects on bacterial adhesion and biofilm formation [4,28,35,36,37] (Table 1), which could be explained by differences in bacterial species and Mg^2+^ concentrations used in the various studies. Since EPS possesses an anionic nature, it was proposed previously that certain Mg^2+^ concentration might contribute to an increase in exopolysaccharide (EPS) production and biofilm stabilization [38]. It was also reported that Mg^2+^ limitation is an important environmental trigger of *Pseudomonas aeruginosa* biofilm development [39]. However, it was found that biofilm formation decreased with increasing concentration of Mg^2+^ in *Enterobacter cloacae* [40]. Moreover, another recent study demonstrated how Mg^2+^ ions affected *Bacillus subtilis* biofilm formation by down-regulating the expression of extracellular matrix genes by more than 10-fold [4]. Taken together, the literatures up to now suggest that, in low concentrations, Mg^2+^ ions seem to induce adherence of bacteria to surfaces and subsequent biofilm formation, while higher concentrations seem to reduce the biofilm formation.

**Figure 1:**
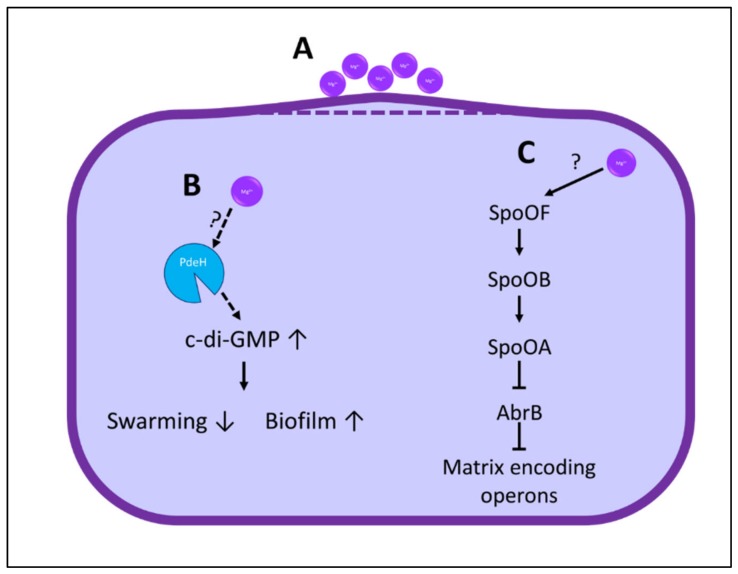
Possible mechanisms for the influence of Mg^2+^ ions on biofilm formation. **A**: Mg^2+^ can affect the membranes curvature, which results in a more sensitive bacterial population. **B**: Elevation of c-di-GMP levels leads to inhibition of the swarming motility and increased biofilm formation. The activity of PdeH, the enzyme that degrades c-di-GMP, is Mg^2+^ dependent. Therefore, Mg^2+^ ions could possibly enhance c-di-GMP degradation and hence decrease biofilm formation that results in heat sensitive bacteria. **C**: A third possible explanation is that the Mg^2+^ ions directly regulate the pathway leading to biofilm formation, which would again result in heat sensitive bacteria.

Thus, magnesium ions have a reasonable potential in affecting the food associated biofilm formation and by this preventing food spoilage and losses in the food industry. The exact mechanism as to how exactly the magnesium ions operate and delay biofilm formation remains unclear, yet several suggestions arise [5,11,41,42] (Figure 1). They could directly interact with the membrane and in some way prevent biofilm formation. Alternatively, they could also directly or indirectly influence the regulation of biofilm formation and delay biofilm formation. Due to the promising results obtained with magnesium ions in prevention of biofilm formation, the effect of Mg^2+^ ions on biofilm formation in the context of food matrices has also been recently studied.

### 2.2. The Effect of Magnesium on Microbiological and Technological Properties of Milk

**Figure 2:**
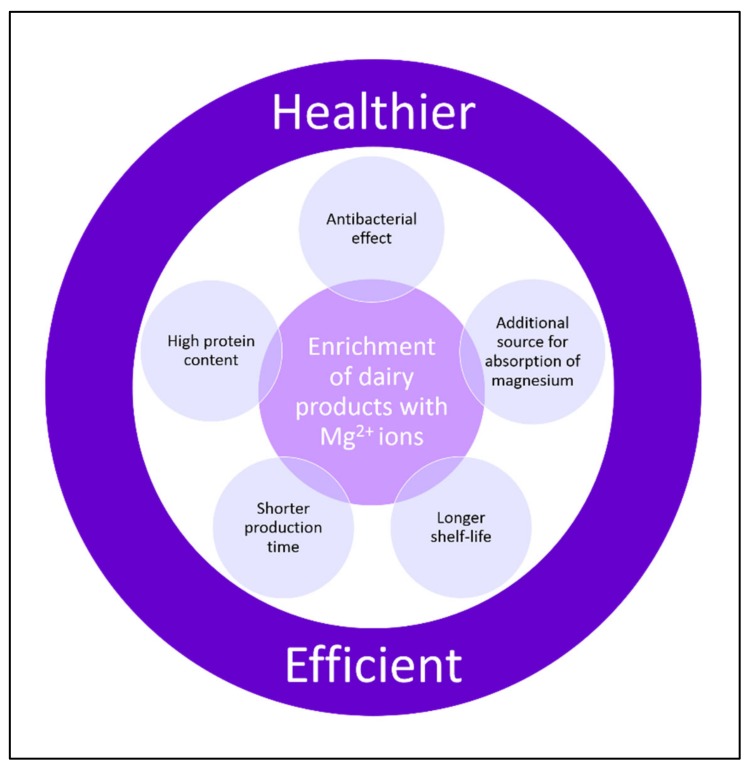
Enrichment of food products, for instance, dairy products, with magnesium would provide pronounced advantages and eventually result in healthier and efficient food products.

Milk is highly nutritious as it contains abundant water and nutrients, such as lactose, proteins, and lipids, and has a nearly neutral pH. This makes it an ideal medium for the growth of different microorganisms. Since microorganisms in milk may hold spoilage and health risks, milk manufacturing is subject to extremely stringent regulations. These regulations include pasteurization at high temperatures, which kills most bacteria, and milk storage at low temperatures, which limits the growth of many bacteria. It has been shown that in several *Bacillus* strains, milk triggers the formation of biofilm [43], and this might make the bacteria more resistant to pasteurization. A recent study has shown that supplementation of milk by 5mM MgCl_2_ and above is capable of impairment of biofilm formation [44]. The impairment of the biofilm eventually results in about a two-log reduction in survival rate of bacterial cells once exposed to heat-pasteurization [44]. Accordingly, enrichment of milk and its products with magnesium would eventually result in safer dairy products as well as this would enable a longer shelf life of the products. In addition, enrichment of food with Mg^2+^ ions may also influence its technological properties as well [44,45]. It was also suggested that in the presence of Mg^2+^ ions the milk clotting starts significantly earlier, and the obtained curd is notably firmer [44]. This finding indicates that the curdling process appears to be improved in the presence of Mg^2+^ ions; i.e., in order to obtain cheeses in a desired hardness, the curdling process in the presence of Mg^2+^ ions is shorter. In another study in which magnesium lactate was added to fat free milk to produce yogurts, the hardness of the yogurts was increased [45]. Moreover, it was also demonstrated that fortified cheeses with Mg^2+^ ions had higher protein quantity [44]. Therefore, enrichment of milk with magnesium not only makes the dairy products healthier, but also improves their technological properties and increases potential availability of this essential mineral for absorption from the magnesium-enriched products. Taking into account also the antimicrobial effect of magnesium, which results in longer shelf life, enrichment of food with magnesium would result in healthier and inexpensive food (Figure 2).

## 3. The Existing Need for the Enrichment of Food with Magnesium

Magnesium is a vital mineral that takes part in hundreds of enzymatic activities, and consumption of a sufficient amount magnesium is highly important for human health. This vital cation also plays important roles in the physiological functioning of the brain, heart, and skeletal muscles and has anti-inflammatory properties. Low levels of magnesium are associated with a wide range of diseases such as migraine, Alzheimer’s disease, hypertension, insulin resistance, pre-eclampsia and cardiovascular diseases [1,46]. The recommended daily allowance of Mg^2+^ according to the US Food and Nutrition Board is 420 mg for men and 320 mg for women. However, it is estimated that most people do not consume the recommended daily allowance of magnesium [2,47,48], and about 10% to 30% of a given population are in a condition of Mg^2+^ deficiency (MGD) [49]. MGD as a result of low intake of magnesium could potentially increase risks for various diseases. Hence, finding new means to supply magnesium to humans is essential. According to [44] an evaluation of the bioavailability potential of magnesium in milk enriched with 5–10 mM MgCl_2_, is ~75–90 mg/L. Hence, one needs to consume over 3.5 L of fortified milk to reach the lower limit of the recommended daily allowance. Nevertheless, consumption of milk fortified with Mg^2+^ will enable to increase the daily consumption of magnesium. Enrichment of food products with magnesium may provide a novel mean to deliver this important mineral to humans and other mammals [50].

## 4. Discussion

As mentioned above, magnesium plays a vital role as a cofactor in numerous enzymatic reactions in the cell [1,2,51]. These include phosphorylation and catalytic reactions, carbohydrate metabolism, lipid metabolism, as well as protein and nucleic acid synthesis. It also plays a role in the active transport of calcium and potassium ions across cell membranes. This vital cation is highly important for the human health [2,52,53]. Here, we elaborated on magnesium in the aspect of its antimicrobial effects. At high concentrations, magnesium ions decrease adherence of bacteria to surfaces and impair biofilm assembly. This makes the bacterial cells more sensitive to heat treatments. In dairy products, lower concentrations (~5mM) are required, maybe due to additional antimicrobial molecules found in the milk. Therefore, addition of magnesium ions to food and especially to dairy products would result in safer food with a longer shelf-life. Moreover, the magnesium ions also improve the technological properties of the products, which eventually results in cheaper production costs. Most importantly, enrichment of food products with magnesium ions would enable a new efficient source of consumption of this important mineral.

## 5. Conclusions

Magnesium is a vital mineral, which is not consumed to a sufficient quantity. Addition of magnesium to food matrices, for instance, to dairy products has several added benefits. First, the antibacterial effect of Mg^2+^ ions enables development of the safer and healthier food. Second, improvements in the technological properties of the magnesium supplemented food enables shorter production time and high protein content of the food products. Finally, enrichment of the food with Mg^2+^ ions provides a new source for the delivery of this vital mineral to humans.

## Figures and Tables

**Table 1:** Varying effects of magnesium ions on bacterial adhesion and biofilm formation.

Bacteria	Influence of Magnesium Ions	Reference
*Staphylococcus aureus*	High concentrations of magnesium bind TA, which increases cell wall rigidity and results in better adherence.	[30]
*Pseudomonas aeruginosa*	Adherence of two of three tested *P. aeruginosa* strains was enhanced by magnesium ions	[32]
	Magnesium ions limitation represses the expression of retS which leads to increased aggregation, exopolysaccharide (EPS) production and biofilm formation	[36]
	Diverse effect of divalent ions on *Pseudomonas aeruginosa* strains of various origins	[38]
*Staphylococcus epidermidis*	Adherence of all tested strains was enhanced in low concentrations of magnesium	[31]
Group b streptococci	Magnesium had no effect on adherence at physiologic concentrations	[33]
*Pseudomonas fluorescens*	Magnesium ions increased initial attachment and altered subsequent biofilm formation and structure	[24]
*Bacillus* species	Magnesium ions are significantly inhibited biofilm formation of Bacillus species at 50 mM concentration and higher. The expression of the two matrix operons was reduced drastically in response to magnesium ions	[34]
	Fortification of milk with magnesium mitigated biofilm formation by *Bacillus* species	[39]
*Enterobacter cloacae*	Biofilm formation decreased with increasing concentration of magnesium ions	[37]
*Arthrobacter* sp.	Mg^2+^ induced biofilm development through the removal of toxic hexavalent chromium	[40,41]
